# Understanding the Immune System in Fetal Protection and Maternal Infections during Pregnancy

**DOI:** 10.1155/2022/7567708

**Published:** 2022-06-24

**Authors:** Tarique Hussain, Ghulam Murtaza, Dildar Hussain Kalhoro, Muhammad Saleem Kalhoro, Yulong Yin, Muhammad Ismail Chughtai, Bie Tan, Anjaleena Yaseen, Zia Ur Rehman

**Affiliations:** ^1^College of Animal Science and Technology, Hunan Agricultural University, Changsha, 410128 Hunan, China; ^2^Animal Sciences Division Nuclear Institute for Agriculture and Biology College, Pakistan Institute of Engineering and Applied Sciences (NIAB-C, PIEAS), Faisalabad 38000, Pakistan; ^3^Department of Animal Reproduction Faculty of Animal Husbandry and Veterinary Sciences, Sindh Agriculture University, Tandojam, Sindh 70050, Pakistan; ^4^Department of Veterinary Microbiology Faculty of Animal Husbandry and Veterinary Sciences, Sindh Agriculture University, Tandojam, Sindh 70050, Pakistan; ^5^Department of Animal Products Technology Faculty of Animal Husbandry and Veterinary Sciences, Sindh Agriculture University, Tandojam, Sindh 70050, Pakistan; ^6^Institute of Subtropical Agriculture, Chinese Academy of Sciences, Changsha, 410125 Hunan, China; ^7^College of Veterinary Sciences Faculty of Animal Husbandry and Veterinary Sciences, The University of Agricultur, Peshawar 25120, Pakistan

## Abstract

The fetal-maternal immune system determines the fate of pregnancy. The trophoblast cells not only give an active response against external stimuli but are also involved in secreting most of the cytokines. These cells have an essential function in fetal acceptance or fetal rejection. Other immune cells also play a pivotal role in carrying out a successful pregnancy. The disruption in this mechanism may lead to harmful effects on pregnancy. The placenta serves as an immune barrier in fetus protection against invading pathogens. Once the infections prevail, they may localize in placental and fetal tissues, and the presence of inflammation due to cytokines may have detrimental effects on pregnancy. Moreover, some pathogens are responsible for congenital fetal anomalies and affect almost all organs of the developing fetus. This review article is designed to address the bacterial and viral infections that threaten pregnancy and their possible outcomes. Moreover, training of the fetal immune system against the exposure of infections and the role of CD49a + NK cells in embryonic development will also be highlighted.

## 1. The Immune Function of the Important Organs

The immune function of the important cells and organs are discussed in the following sections.

### 1.1. Trophoblast Cells

Trophoblast cells originate from a blastocyst and seem to appear four days after fertilization in humans [1]. Its function is to supply nutrients to the embryo and develop into a key part of the placenta [2]. The trophoblast cells recognize blastocyst contact with maternal decidua. These cells regulate the molding in the immune system around the implantation site and give an active immune response against external stimuli. The trophoblast cells secrete most common cytokines such as chemokine ligand (CXCL12 and CXCL8), transforming growth factor (TGF), and the chemokine ligand 2 (CCL2). These cells also encourage the recruiting of peripheral monocytes, neutrophils, natural killer cells (NKs), etc. to the binding site of implantation [3]. Once the immune infiltration occurs after decidualization, the rush of the immune cells is pivotal for normal gestation. Disruptions in this mechanism impede immune infiltration and eventually cause detrimental effects on pregnancy outcomes [4].

Trophoblast triggered cytokine production to stimulate immune cell recruitment and differentiation, giving them a phenotype for a successful pregnancy [3]. The decidual natural killer cells (dNKs) are differentiated from peripheral natural killer cells (pNKs), responsible for the production of interleukin-15 (IL-15) (trophoblast cells) and transforming growth factor-beta-12 (TGF*β*12). These particular NKs are essential for decidual vascular remodeling and placental function [1]. The cluster of differentiation-14 protein (CD14)-positive monocytes gain a distinctive M2-like macrophage phenotype around maternal-fetal interface, which is supposed to be augmented by trophoblast-induced macrophage colony-stimulating factor (M-CSF) and IL-10 [2, 3]. These macrophages are involved in tissue remodeling, degradation of extracellular matrix [4], and apoptotic cells. The M2-like macrophages also maintain CD14 expression while secreting cytokines (TGF*β* and type-I interferons) [5]. TGF*β* is produced from trophoblast cells and triggers the variation of naive CD4^+^ cells to forkhead box P3 (FOXP3) and positive Treg cells [6, 7]. Previous evidence implies that decidual cells, like trophoblast cells, influence diverse immune cell functions around the implantation site [8].

Trophoblast cells actively respond to expressions of chemokines and cytokines that may attract and educate immune cells. Toll-like receptors (TLRs) and Nod-like receptors (NLRs) respond to innate immune sensors, which provide quick responses against pathogenic invasion or tissue injury [9] from bacteria, viruses, and other microbes [10, 11]. Hence, it permits trophoblast cells to analyze and respond against these particular signaling molecules. In summary, trophoblast cells train immune cells and give signaling responses in a unique way that helps in performing several functions of fetal growth and development [12].

### 1.2. Embryo Protection

Previously, emphasis was given to embryo protection against microbial infections and congenital consequences of certain infections [13, 14]. The placenta acts as an immune barrier that defends the fetus from invading pathogens. The syncytiotrophoblasts (SYNs) cells form the barrier between maternal and fetal blood [15]. After differentiation, SYNs are greatly strong to viral infection and unable to express the recognition receptors of viral pathogens including herpes simplex virus (HSV) and cytomegalovirus (CMV) and also possess a cytoskeleton network that helps them out from Listeria monocytogenes [14, 16]. SYNs also release exosomes and type III IFNs (IFN-*λ*) that confirm antiviral ability in and to adjacent cells [15, 17].

Pathogens can also get into the fetus through the uterus [18]. Various immunoprotective strategies are required to avoid the pathogen's route. Effector-memory CD8-positive T cells exist in human endometrium, but few of them are pathogen-specific [19]. These cells are less cytotoxic compared with peripheral counterparts; dNKs enabling the killing of HCMV infected cells and decidual CD8 positive T cells may degranulate and multiply followed by in vitro stimulation [20]. The mechanism by which a few pathogens penetrated the fetus instead of these barriers is unclear. Another aspect of fetus protection is the maternal immune system. Maternal IgG antibodies are transferred to fetus through the neonatal Fc receptor present over the SYNs [21]. In humans, IgG transfers to the fetus during the second stage of pregnancy [22]. In vivo investigations from the placenta have shown that there is no indication of transplacental transmission of many cytokines [23]. The prolonged maternal infection with human immunodeficiency virus 1 (HIV-1) or hepatitis B may result in increased production of cytokines in fetal blood that modify fetal immune response suggesting that the maternal immune system affects the fetus via the production of placental cytokines [18]. Moreover, maternal IL-17 may influence fetal brain development [19]. It has also revealed that the developing embryo has anti-infection properties. Pluripotent stem cells, particularly embryonic stem cells, have been shown to have antiviral properties due to the presence of continuously expressed interferon genes (ISGs), such as interferon-induced transmembrane protein-1 and 3 (IFITM1) and IFITM3 [24]. At the same time, developing embryos defend themselves from exogenous pathogens as well as endogenous genomic deleterious effects [25].

### 1.3. Fetal Immune Response

It has been recognized that a fetus is greatly vulnerable to infections, specifically in the first phase of pregnancy because of diverse stimuli. Therefore, the growing fetus is relying on an innate immune response to microbial infection [26]. Dasari et al. documented that TLRs are present on neonatal monocytes and granulocytes same as adults. Moreover, the phagocytic property of NK cells, macrophages, and dendritic cells (DCs) has been reported same as in adults but with a low antigenic response [27]. Considering the several studies on ZIKA virus, Chen et al. has reported that fetus release type-1 interferon (IFN-I) signals involved in anti-ZIKA virus response and this molecule contributed to antiviral activators (JAK1 & TYK2) and (STAT1 & STAT2), which in turn activate several genes related with IFN-stimulated genes (ISGs) [28]. Recent investigations indicate that maternal transferred fetal immunity is not strong but it becomes solid after 22 weeks of gestation; it enhanced IgG level and increase maternal level around the birth process [29].

The lung, gastrointestinal tract, and skin of the fetus are susceptible to infection during pregnancy. When the skin is infected, epidermal keratinocytes release cathepsin, a peptide that suppresses bacterial growth or destruction, resulting in enhanced volume [30]. It has shown that chorioamnionitis induced increased expressions of TLR2 and TLR4 as well as cytokines, chemokines, and other factors [31, 32]. The immune cells in the lung are alveolar macrophages, and chorioamnionitis may increase the formation of these cells through fetus immune response. Stimulation of IL-6 causes secretion in the placenta, not involved in type II alveolar cells but also triggers SP-A induction in the maturation of lungs, therefore increasing further fetal lung immunity [33]. The first layer of the gastrointestinal defense is microfold cell. The lamina propria consists of diverse immune cells such as DCs and macrophages in the intestinal epithelium [34]. The fetal intestinal epithelial cells are susceptible to lipopolysaccharide (LPS); its induction releases a cytokine, IL-8, that recruits more immune cells, which are responsible for the intactness of the immune barrier [35].

### 1.4. The Placental Barrier Function

The placenta has been shown to exert diverse functions in pregnancy, including exchanging gases, nutrients, metabolites, and hormones within maternal and fetus and also serves as an unsusceptible function barrier [36]. Toll-like receptors are present over mononuclear macrophages, lymphocytes, and epithelial cells [37]. These receptors are regularly expressed on the placenta. The receptors of TLR2 and TLR4 exist in placental villi and trophoblast [38, 39]. The expression of TLR2, TLR3, and TLR4 is mostly declined during early pregnancy [40]. It shows that stimulation of TLRs in the placenta may possess several functions, consisting of immune cell recruitment, cytokine production, and defense against infections [41].

Multipotent trophoblast progenitor cells (TBPCs) were observed to localize in the human placenta (chorion) and distinguish into mature trophoblast subtypes that eventually form the functional placenta [34]. The increased immune activities of trophoblast cells around maternal-fetal interface are non-repairable due to recruitment of immune cells against bacteria and virus infections [35]. The evidence has confirmed that trophoblast cells identify pathogen utilizing different TLRs and then secretes cytokines and chemokines, which is responsible for removing infectious pathogen [42]. Epithelial cadherin (e-cadherin), a receptor, is localized in the cytotrophoblast layer that may conserve Listeria endotoxin A to confine the scattering in the bacterium [43]. The viruses such as cytomegalovirus (CMV) and Porphyromonas gingivalis and the trophoblast cells may bind to the TLR3 receptor, to enhance the production of SLPI and IFN-*γ* to viruses which result in avoiding the spread of virus towards the placenta and fetus [44]. However, the decidual trophoblasts may produce CXCL12 (SDF1), CXCL8 (IL-8), TGF-*β*1, and CCL2 (MCP1) to recruit macrophages, NK cells, and regulatory T (Treg) cells, indicating a relationship between innate and acquired immunity [12].

**Figure 1:**
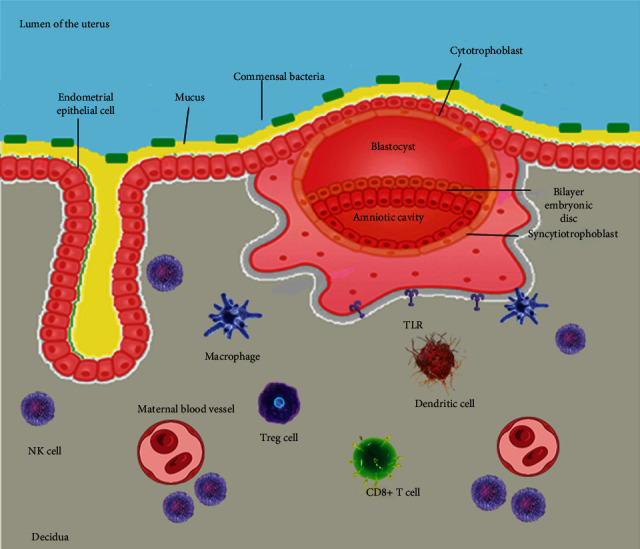
The immune cells around maternal-fetal interface figure courtesy, [130].

Fetal syncytiotrophoblasts develop a unique surface that pours into the maternal blood close to the cytotrophoblast layer. When syncytiotrophoblasts are threatened by infections in the maternal blood, these cells exert different mechanisms against T. gondii, Listeria monocytogenes [45], ZIKV, HSV, and CMV viruses that may involve in lacking receptors [16]. The syncytium's surface possesses distinct physical characteristics having dense branches microvilli and a complex actin network [45]. Moreover, syncytiotrophoblasts have a younger index than red blood cells in anemic subjects suggesting a hard level that avoids microbial penetration through the trophoblast layer [16]. In addition, maternal blood macrophages are bound towards microorganisms; later, they yield 2,3-dioxygenase, *β*-defensins, ROS, etc., enabling them to get entry in trophoblast resistance against infections [46]. Listeria infection may defend syncytiotrophoblasts in the first trimester of pregnancy that is regulated via the transportation of placental exosomes carrying miRNA and IFNs [47]. The immune cells present at maternal-fetal interface are shown in Figure 1.

The placenta, itself only bears an acquired immunity. It was reported that maternal CD4+ T cells display a key part in governing maternal immunity to fetal death. The formation of CD4+ T-cell cytokines and interface among CD4+ T cells and antigen-presenting cells activate proliferation of the cytotoxic CD8+ T-cell population; thereafter, cytotoxic cells may clear utilizing Fas/Fasl pathway [48]. Furthermore, CD4+ and CD8+ T cells are involved in Toxoplasma infection [49]. Another study conducted by Goldberg et al. revealed that there are eight complement of cytokines present in villi namely factors B, C3, C1r, C1s, and C1 and the suppressing factors H, C4, and C2 [50]. Furthermore, the investigation highlighted that C3 and C4 primarily appear in trophoblast cells while the IFN-*γ* may enhance their expression. The secretion of these molecules enhances the defense in placental function [35].

## 2. Immune Response to Microbial Infections

As aforementioned, trophoblast cells regulate immune response at the maternal-fetal interface, to promote tolerogenic phenotype. It can sense and respond to receptors presence on microorganisms. Vulnerability to infection may compromise the immune system around maternal-fetal interface resulting in pregnancy problems including chorioamnionitis and premature delivery [42, 51]. In 40% of preterm delivery cases, bacterial infections have been diagnosed [43], while 80% of premature births occur before the 30^th^ week of pregnancy, suggesting evidence of infection [52]. Bacterial infections can enter the maternal-fetal interface via three different routes: ascending, descending, and maternal blood circulation [53, 54]. After penetration in placental and fetal tissues, the bacterial infection is considered a risk to pregnancy and fetus. It triggers an immune response against a pathogen that may promote inflammation destroying fetal and placental cellular constituents [55, 56]. It has been documented that trophoblast and immune cells can improve fetal acceptability; however, overactive response of these cells to bacteria resulting to fetal rejection [57, 58]. Animal studies have demonstrated that bacterial components contribute to preterm birth [59, 60] and in the presence of placental infection and inflammation [61]. Similar studies have been conducted in clinics, and evidence shows that preterm delivery linked to placental infection and inflammation.

The maternal microbiota is known to be involved in immune tolerance and anti-inflammation response, which is evident in the second stage of pregnancy. Trophoblast cells possess the capability to respond against pathogens through the expression of highly conserved receptors [62]. It was observed that LPS triggers respond via TLR4 in mouse, human trophoblast cells, and trophoblast-educated macrophages; it bypasses NF-*κ*B triggered inflammation but produces type I IFNs. This interferon's which include IFN*α* and IFN*β* are polypeptide in nature, exerting three functions such as antimicrobial response, controls of innate immune response, and stimulation of adaptive immune response [63]. The production of IFN*β* has been linked with placental tissues in many species including humans [64], an elevated amount indicates that in addition to antiviral activity, and IFN*β* may influence inflammatory response by TLRs [63, 65]. IFN*β* is also the primary modulator of immunological response during pregnancy, as evidenced by IFN receptor-deficient mice. LPS induction in wild-type animals has reported normal pregnancy outcomes; IFN receptor-deficient mice were documented to enhance LPS sensitivity and cause preterm birth within 24 hours. The lack of IFN receptors was linked to the production of cytokines and chemokines such as TNF, IL-6, and IL-8, all of which have been connected to preterm birth induction [66].

### 2.1. Bacterial Infections in Pregnancy

Bacteria-induced intrauterine infection triggers the formation of pro-inflammatory cytokines which shows an important role in preterm labor. Many of the microbes get access to the uterus through the female reproductive tract when insufficient defense of the cervix and mucosa of the reproductive tract [67]. Another way of the infection is by the maternal circulation; it has been documented that bacteria (Fusobacterium nucleatum), which contributed to peripheral infections [68], may be identified in amnion and may trigger inflammation and pregnancy problems. When the pathogenic bacteria get accessed to decidua, inflammation is prevailed by stimulation of specific receptors and cytokines, which may influence pregnancy outcomes such as preterm birth and affect fetal growth. For instance, an inflammatory cytokine, IL-1, has been involved in women having preterm labor; it appeared in human decidua against bacterial endotoxin, but also triggers prostaglandin production via decidua and may contribute to myometrial contractions [55]. In addition, TNF-*α*, a pro-inflammatory cytokine, has been reported to be enhanced in amnion of preterm women [69], and it is upregulated in response to bacterial products in women and animal models [70]. Thus, it shows that TNF-*α* may involve in pregnancy problems via increasing prostaglandin production and myometrial contractions, but it may induce premature cervical ripening through upregulation of matrix metalloproteinases [59].

Intriguingly, it was thought that the uterus and the amniotic cavity were deliberately sterile, though the new findings using molecular tools have revealed that bacteria were found in fetal membranes of up to 70% of women experiencing cesarean sections at term [60, 61]. Moreover, sequencing data have observed the presence of “placental microbiome” in normal pregnancies. It advises that the prevalence of bacteria itself is not pathogenic and is needed to influence normal function. For further evidence of bacterial infection during pregnancy in mice, please refer to this article [71].

### 2.2. Viral Infection during Pregnancy

A small number of epidemiological evidence has suggested a link between viral infection and preterm birth and fetal anomalies [72], although it has been well-known that pregnant women are vulnerable to a few viral infections like influenza A virus, hepatitis E virus (HEV), and herpes simplex virus (HSV) [73] than the non-pregnant ones. To explain viral infection in pregnancy, an animal model has been utilized for subclinical viral infection [71]. During placental infection; itself, not cause preterm birth, but induce developmental anomalies of fetal brain and lungs [74]. Intriguingly, murine herpes virus models have been reported to cause viral-associated perinatal neurologic injury in the USA [75]. This pathology prevails probably in the first phase of maternal pregnancy or infection around delivery, although irrespective of placental transmission, the fetus might be influenced via maternal infection [76].

The placental infection of the virus induces mild inflammation and is unable to terminate the pregnancy, but may trigger the immune function from both sides: the mother and the fetus. Thus, it has numerous outcomes; for example, it triggers inflammation in a fetus in absence of the virus. It is the so-called fetal inflammatory response syndrome (FIRS) and is classified non-existence of cultivable microorganisms; however, placental infection induces an increased level of inflammatory cytokines such as IL-1, IL-6, IL-8, and TNF-*α* [77, 78]. These molecules have been demonstrated to influence CNS and circulatory systems [78, 79] and may result in fetal morphologic anomalies in animal models consisting of ventriculomegaly and hemorrhage. Further, the prevalence of FIRS has been linked to higher risk of autism, schizophrenia, neurosensorial deficits, and psychosis in later stages of life [80, 81]. The other vulnerability of viral infection to placenta is to sensitivity against bacterial co-infection. The adverse outcome of viral infections during pregnancy is illustrated in Table 1.

### 2.3. Outcome of Pregnancy Infections

Maternal infection in pregnancy with microorganisms induces inflammation and eventually causes fever, diarrhea, and abdominal pain. The presence of inflammation during pregnancy delivers adverse outcomes for example increase risk of miscarriage, premature birth, and stillbirth [82]. After penetrating the virus into fetus, it triggers inflammatory response due to cytokines that may influence fetus brain development and circulatory system and causes risk to schizophrenia, autism, and mental disorders [83]. Furthermore, nearly half of pregnant women who had an infection despite no symptoms may deliver birth prematurely, which has been linked to previous placental infections (acute and chronic chorioamnionitis) [84].

Studies on epidemiological and microbiological indicate that 25–40% of premature births occurred due to intrauterine infection [85]. Notably, a threat to microbial infection in pregnancy is not linked to pregnancy problems but influences different infant organs. For instance, rubella virus (RV), cytomegalovirus (CMV), herpes simplex virus (HSV), *Toxoplasma gondii*, and other viruses may result in premature birth, stillbirth, and even neurological disorders after birth [13, 86]. Moreover, the infection of microbial risk in fetuses and disease severity depends upon the pregnancy stage. The example includes infection of RV in the first phase of pregnancy that can induce abortion and genetic anomalies, although, when pregnancy approaches mid and late stages, the incidence of genetic malformation due to RV is very low. However, the third phase of pregnancy is most vulnerable to CMV infection but huge damage prevails in the first phase of pregnancy. The abovementioned processes might be influenced due to variations in growth, stage of fetus development and the resistance against external stimuli in various stages of pregnancy [86].

Maternal infection in pregnancy may pursue diverse health ailments in the fetus following birth. An investigation conducted by different organizations revealed that gut microbes are also involved in fetal autism. The study further indicated that intestinal microbial infection in pregnant women may trigger immune cells to produce the huge level of IL-17 that gets through the placental barrier into a fetus, creating plagues in the S1DZ region of the fetal brain. As in turn, the fetal central nervous system develops autism [87].

## 3. Mechanism of Immune System in Regulating Fetal against Microbial Infection

### 3.1. Prenatal Exposure to Infection Shapes Early Immunity

Previous studies propose that maternal exposure to noninfectious and infectious microbes forms the fetal and later neonatal immune response. The widely studied maternal immune system to fetal and neonatal immunity is the transfer of immunoglobulin's from mother to fetus. The transfer may occur either through the placenta or breast milk, regulated by the neonatal Fc receptor, FcRN [88], and delivers major protection to the newborn. Notably, maternal IgG antigen transfer through placenta forms complexes by FcRn and may result in antigen specific immune response in fetal cells [89–91]. The FcRN mechanism may underline antigen-specific responses against parasitic antigens by newborn lymphocytes in the response of maternal infection with schistosomiasis, placental malaria, Chagas' disease, and HIV. Of note, that fetal infection, itself, is not the demand for in-utero shaping of fetal immune system [92]. Maternal transfer of antigens may trigger prevalence of antigen-specific Tregs [93], though this maternal produced antigen-specific fetal Tregs which are derived from fetal thymus (nTregs) or periphery (pTregs) is not clear [94].

Several studies propose that fetal immune system may be trained in pregnancy [95, 96], through which maternal infection triggers systemic modifications in fetal immune system. The best evidence of infants born exposed to but infected with HIV [97]. In utero exposure, without vertical transmission of HIV, results in increased neonatal cytokines profiles of monocytes triggered with diverse TLR agonists [98]. Likewise, infants get exposed to malaria was reported to reduce the low level of innate cytokines in cord blood, though, the increased response to activating specific TLR agonists [99, 100]. The infants of humans exposed to hepatitis B virus (HBV) in utero have increased level of antiviral cytokines in the cord and show strong chances of stimulation and maturity of cytokines [101]. Vaccination of Bacille Calmette-Guérin (BCG) during pregnancy may strengthen the innate immune response in offspring and proinflammatory cytokine in infants exerted by TLR activation [102]. Trains the immune system in infants, which appear without vertical transmission, examine the ability of the fetal immune system to respond in an indirect way to maternal infection or inflammation [103].

### 3.2. Maternal Inflammation Train Fetal Immune System

The underlying mechanism of fetal immune system against fetal infection has not been explored, though the indirect mechanism via exposure of maternal infection is under investigation. The one clarification of fetal immune system could be the maternal cytokines or other inflammatory mediators into circulation, thereafter, activation of fetal immune system. Knowing whether the maternal cytokines cross placenta in human's gestation is tremendously difficult, ex vivo experiments having human placenta propose that cytokine transfer via placenta is limited at later stages of developments [23, 104]. However, studies in rodent models propose that few cytokines cross the placenta during early stage of pregnancy [105, 106] and eventually alters the neonatal immune response towards infection [107]. Dahlgren and his team reported that transplacental transfer of iodine-125 labelled IL-6 was increased at mid-gestation than the late gestation, indicating that immature placenta becomes more permeable to maternal cytokines [105]. Specific pathogen-related TLR ligands were known to be crossed through mouse placenta at mid-gestation directly connect with fetal cells, though the direct effect was not observed [108]. Moreover, other TLG ligands can cross the placenta and straightly exert fetal immune response has not been investigated. Generally, we have less information regarding maternal cytokines which can trigger cytokine productions in fetal side. Lastly, vertical transmitted pathogens activate fetal immune response in utero. More research on this aspect is needed to dig out the role of maternal cytokines in fetal immune response [108].

Another way the fetus might respond indirect against inflammation or interfere with placental function proceed by maternal infection. Chorioamnionitis, a placental infection induced by non-pathogenic microbes, carries systemic modification in fetal immune system, comprising generation of cytokines and lymphocyte divergence [109]. Interestingly, fetal cytokine production has been reported without observable amniotic infection in a macaque model of streptococcal-induced chorioamnionitis [110], showing that the fetus may respond directly to more signals over fetal unit. Viral infection in maternal placenta may generate fetal cytokines in mice irrespective of fetal infection [76]. Current studies on cord blood during preterm human infants propose that inflammation at maternal-fetal interface shapes fetal lymphocytes to generate inflammatory cytokines comprising TNF-*α* and IFN-*γ* in preterm infants [111]. Genetic evidence revealed the involvement of fetal response to placental malaria indicating fetal innate immune signaling to overcome placental malarial infection [112]. Hence, a fetal immune response occurs due to the maternal inflammation, as in contrast or directs response against maternal inflammation.

Irrespective of inflammation and infection, collective studies reveal that maternal microbiome may straightly interfere fetal immune development and function in utero. However, direct transfer of maternal microbes to the placenta of fetus induces fetal demise, while indirect interaction through microbial metabolites may affect fetal immune development. Restricted exposure of E. coli to gestation makes colonization and results in particular alteration in fetal innate immune compartments, for instance, gut type III innate lymphoid cells (ILC3s) and mononuclear cells [113]. Such exposure may rely on maternal antibody-related microbial molecules but may also be transferred through the exposure of microbial metabolites. Short-chain fatty acids (SCFA) derived from microbiota may provision to fetal circulation and affect fetal immune cell production, function, and eventually offspring immunity [114]. Currently, supplementing SCFA during pregnancy has been observed to restore thymic and T-cell developmental defects in a mouse model of pre-eclampsia [115].

### 3.3. Role of CD49a + NK Cells in Embryonic Development

In early pregnancy, the endometrium changes into decidual tissue with the help of estrogen and progesterone, shaping a maternal-fetal interface due to the maternal and fetus interaction [116]. CD49a binds to collagen and laminin and is known to be the biomarker of tissue-resident NK (trNK) cell subsets in mice [103, 117]. The uNKs in humans have huge amount of CD49a + trNK cells, particularly CD49a + Eomes+ uterine trNKs. These cells comprise 85% of all NKs from normal human decidua in first trimester, secrete growth-promoting factors, and thereby increase fetal growth in early phase of the fetal development. Reduction of CD49a + Eomes+ uterine trNK cells interference in the secretion of growth-promoting factors is prevalent in miscarriage patients [118], though uterine CD49a + trNK subsets in menstrual blood may foresee irregular endometrial status [119]. Therapeutic intervention with CD49a + NKs for human pregnancy-related problem might be enabling to attenuate the impact of constrained nourishment within uterine microenvironment.

### 3.4. Function of NK Cells in Fetal Growth and Development

Normal pregnancy is a sensitive process for fetal growth, development, and preservation of immune tolerance. Uterine natural killer cells (uNKs) are the key cells differentiable from lymphocytes in first trimester during pregnancy, organizing >70% of all leukocytes in human deciduas [120]. As already discussed above the uNKs in pregnancy, it reduces once the placenta is shaped. Communications of NK cell–specific receptors and their ligands express either invasive decidual stromal cells or trophoblasts derived uNKs perform several functions including placental growth, decidualization, trophoblast invasion, and immune balance [121]. The angiogenic regulating molecules such as cytokines and chemokines elicits a beneficial effect on placentation and birth weight [122]. The difference among uNKs and fetal growth restriction (FGR) in interleukin15-deficient (IL-15^-1-^) mouse [123] and the transcription factor Nfil3-deficient (Nfil3^-1-^) mouse [124] models, identification of uNKs subset for promoting fetal growth in early pregnancy are missing. It has been known that fetal body weights are co-related with prevalence and function of uNK cells. The cross talk within active KIR2DS1 and HLA-C2 receptors has a beneficial impact on birth weight, while communication among suppressor receptors KIR2DL1 with HLA-C2 has a negative effect on birth weight [125, 126]. uNK cells have long been recognized as dedicated immune cells capable of angiogenic and regulatory properties which establishes during the evolution of pregnancy. It is still unclear whether these transient NKs coordinate in the early optimization of maternal nourishment of the fetus. In a report, uNKs are key regulator cells but are unable capacity to destroy cells around maternal-fetal interface [127–129]. Further, the role of NKs in fetal development the through the secretion of growth-promoting factors is well described by [118].

## 4. Conclusion

Normal pregnancy is a sensitive and very complex immune process which regulates successful pregnancy. Trophoblast cells, immune cells, and placenta are the natural protective system, which keeps fetus safe from internal and external stimuli. However, pregnancy is threatened by several infections like bacteria and virus which causes damage in placental and fetal tissues, triggers inflammatory response due to production of cytokines, and eventually causes fetal rejection. There are also different routes in the body by which fetal immune system is broken down which terminates in fetal damage. Some infections which cause congenital fetal anomalies make fetal life questionable for productive performance. For better understanding, animal models should be used to explore the underlying immune system in fetal acceptance or fetal rejection and its congenital malformations.

## Figures and Tables

**Table 1:** Pregnancy complications linked to viral infections.

Virus	Impact on pregnancy	References
Cytomegalovirus	Congenital hearing loss, neuronal malformation, and intrauterine growth restriction	[48, 131]
Varicella zoster virus	Hypoplasia and premature birth	[132–134]
Rubella virus	Stillbirth, fetal growth restriction, and fetal infection	[135, 136]
Herpes simplex virus	Neurological deficits, blindness, and seizures	[137, 138]
HIV	Vertical transmission of the virus	[139, 140]
Hepatitis A	Miscarriage, preterm birth, and stillbirth	[141, 142]
Hepatitis B	Miscarriage, preterm birth, and stillbirth	[143, 144]
Hepatitis C	Miscarriage, preterm birth, and stillbirth	[145, 146]
Hepatitis E	Miscarriage, preterm birth, and stillbirth	[147]
Ebola virus	Spontaneous abortion and fetal loss	[148]
Lassa virus	Abortion, stillbirth, and fetal death	[149]
Influenza virus	Preterm birth, small-for-gestational-age birth and congenital malformation	[150]
Zika virus	Microcephaly	[151]

Table courtesy Gil Mor et al. [14].

## Data Availability

Access to data is restricted.
